# Exercise-dependent BDNF as a Modulatory Factor for the Executive Processing of Individuals in Course of Cognitive Decline. A Systematic Review

**DOI:** 10.3389/fpsyg.2017.00584

**Published:** 2017-04-19

**Authors:** Gilmara G. de Assis, Katie Moraes de Almondes

**Affiliations:** Department of Psychology and Postgraduate Program in Psychobiology, Federal University of Rio Grande do NorteNatal, Brazil

**Keywords:** aerobic exercise, BDNF, executive functions, cognitive decline, aging

## Abstract

**Background:** Aging naturally triggers a decline in cognition as result of deterioration in cerebral circuits, thus the executive functions (EFs) suffer changes that progress from mild to severe states of impairment. Exercise instead, works as a strategy for cognitive enhancement by modulating neuronal plasticity through the regulation of BDNF. However, whether the exercise-dependent BDNF may improve higher complexity processes such as the EFs is still in a studying process.

**Results:** Current data on exercise-dependent BDNF changes for aging individuals in a course of cognitive impairment was summarized to investigate whether the exercise regulation of BDNF is effective to pronounce long term changes on executive controls. While the exercise-dependent regulation of BDNF is currently undeniable, the role of exercise dependent BDNF as a tool for the improvement of EFs in individuals with dementia is still less clear and seldom discussed. The summary of findings indicate a limited number of studies addressing exercise in order to discuss parameters related to either BDNF or executive functioning in such population conditions (*n* = 215), further narrowing to a total of 5 studies presenting analysis of both parameters. Nonetheless, positive outcomes from BDNF and EF variables were displayed by all the populations exposed to exercise across studies. Aerobic exercise was shown to be a major source for the enhancement of the BDNF-dependent executive functioning, when compared to cognitive stimulation. Moreover, the effect of exercise-dependent BDNF on domains of executive functioning appears to occur in a dose-dependent manner for the aging individuals, independently of cognitive condition.

## Introduction

Unavoidable changes of the aging nervous system are one of the great challenges of modern neurology and medicine. Aging naturally triggers a performance decline in a range of tasks that require demands of cognition with more than 40% of population above 60 years affected by varying degrees of cognitive impairment (Konar et al., 2016). Functional limitations eventually emerge with the hypo-function of neuronal pathways, as a consequence of the losses in brain circuitry on frontal, parietal, and temporal areas. Therefore, processes as perceptual speed, attention, reasoning, learning, and memory display changes that state from mild impairment to severe dementia (Sofi et al., 2011).

Dementia also denotes different shapes and progressions of cognitive decline. The term “dementia” refers to a state of severe acquired intellectual deterioration which significantly interferes with the fulfillment of personal and social roles in a way that ultimately requires care and supervision. It is when the individual presents defects in more than one domains of cognition with sufficient decline to cause executive disabilities that interfere in daily functions (Finney et al., 2016).

From a healthcare perspective, aerobic exercise (AE) holds an important role in modulating anti-inflammatory function and the expression of brain-derived neurotrophic factor (BDNF) (reviewed in Huang et al., 2014; Dinoff et al., 2016) in regions that are critical for cognition, such as the hippocampus, frontal cortex, and fronto-parietal network. BDNF-related improvements in cognition have provided support for the development of newly exercise-based strategies toward the postponement and prevention of neuronal degeneration and the age related cognitive loss (Phillips et al., 2014).

Exercising evokes changes in different levels of a human being’s organization, including molecular. These changes – regarding metabolic adaptations – occur in accordance with the exercise volume – intensity, duration, and frequency (Murawska-Cialowicz et al., 2015). Therefore, in spite of the differences on age, sex, fitness conditions, or else the type of exercise, analysis of metabolic alterations during exercise is an appropriate way of controlling the metabolic dynamics of exercise (Coelho et al., 2013). Thus, exercise effects observed for cognition is expected to occur through this integrated machinery that embraces the aerobic energy expense and the synthesis of BDNF as a hub for the promotion of neural plasticity and protection (Rothman et al., 2012).

Called ‘exercise factor,’ BDNF was identified in muscle cells as a sort of exercise-dependent axis for its gene expression on hippocampal tissue. Because BDNF gene expression is modulated by peroxisome proliferator-activated receptor gamma coactivator 1-alpha, a co-transcriptional factor highly responsive to aerobic metabolism imbalance, exercising became a crucial tool for the enrichment of BDNF concentrations and its related improvements on neuronal circuitry and cognitive processing (Wrann et al., 2013). While severe down-regulation of BDNF represents severe damages on executive circuitry, a positive relation between exercise exposure and the levels of BDNF is strongly associated to increases in neuronal plasticity and cognitive ratios (Flöel et al., 2010; Wrann et al., 2013; Karpova, 2014).

The effects of AE on global cognition in aging individuals range from improvements on hemodynamic support to synaptogenesis, all of which are important for changing brain structure and functioning (Heijnen et al., 2016). Nevertheless, at a higher level of complexity the domains of executable competences, also susceptible to a decline under conditions of diminished BDNF, are not quite understood in terms of exercise-dependent BDNF modulation (Muñoz et al., 2016).

Executive functions encompass the arrangement of executable skills, supported by the activity of many different interconnected brain areas, undergoing cognitive processing. They enable us to hold information in mind and properly switch attention in order to better perform a given task or problem (Blair, 2016). Based on the core abilities of inhibitory control (IC), working memory (WM), and cognitive flexibility (CF), from which higher-order skills are built, the EFs are effortful processes required when you have to pay attention if the ‘going on automatic’ is insufficient. Good executive functioning is thus necessary to inhibit ingrained behaviors, to focus attention strategically, and to organize our thoughts in face of a distraction, complexity, and stress, being imperative for our daily living (Diamond, 2014).

Decreased levels of BDNF have been associated with cognitive decline in aging individuals physically active in specific components of memory, but not in those of EF (Komulainen et al., 2008). Moreover, individuals in advanced stages of cognitive decline present less or no responsiveness to exercise regulation of BDNF specifically in competences of the executive domains. This enforces that higher-order skills such as the EFs are represented by domains less sensitive to changes in BDNF concentrations, when compared to processes of lower complexity as memory.

However, systematic activation of brain networks that are involved in executive processing seem to provide loads of cognitive demand sufficient for triggering adaptive changes so that exercise challenges based in the executive control have demonstrated an enhanced BDNF-dependent synaptic reorganization in the specific domains of executive control exercise challenges based on executive control (Hötting and Röder, 2013; Paillard, 2015). Moreover, the cognitive stimulation itself may also be efficient regarding certain gains of EF (Azadian et al., 2016).

In this study, we hypothesize that increments in domains of executable functions due to the exercise regulation of BDNF in aging individuals undergoing cognitive decline depend on greater amounts of exercise-dependent BDNF stimuli than do those of memory and cognitive processing. For that, a systematic review was applied exploring studies using exercise interventions to evaluate changes in parameters of EF and reporting measures of BDNF for aging individuals undergoing processes of cognitive decline.

## Materials and Methods

**FIGURE 1:**
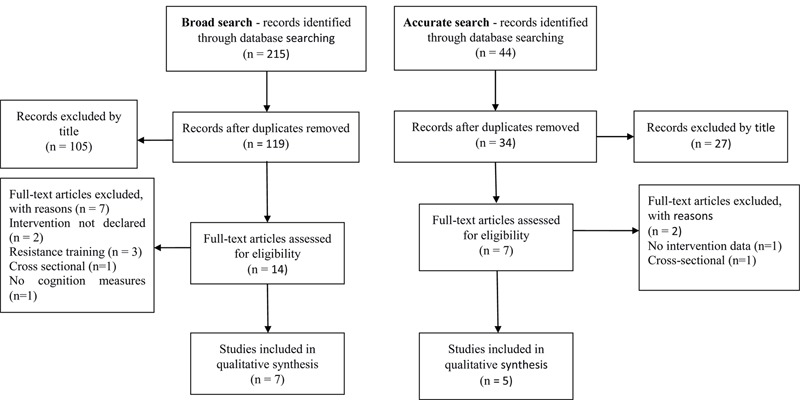
**Search strategies flow chart**.

In order to provide an overview of the existing literature regarding exercise interventions for observing changes in BDNF-related cognition in aging individuals in course of cognitive impairment, we previously searched for a broader strategy of PICO: P- frail elderly OR aging OR mild cognitive impairment OR dementia; I- Aerobic exercise OR Physical exercise; C- N/A; O1- Brain derived neurotrophic factor OR BDNF; O2- Executive function OR cognitive flexibility OR inhibitory control OR working memory OR cognitive function OR cognition. Thereafter, a more accurate strategy was applied to extract the specific outcomes referring to executive processes, relevant for this study – O2- Executive function OR cognitive flexibility OR inhibitory control OR working memory. Searches were approached on PubMed, Scopus and Medline databases. Broad and accurate searches flow chart is in **Figure 1**. Summary of main results is presented in **Table 1**. Details are registered at PROSPERO- ID = CRD42016050017.

**Table 1:** Summary results from searches strategies.

Study	Exercise	Sample	BDNF outcomes	Cognitive outcomes
^∗^Baker et al., 2010	6 months of exercising between 75 and 85% of HR reserve, 45 to 60 min/day, 4 day/week, compared to a stretching control.	33 adults (17 women) with MCI – age between 55 and 85 years.	Aerobic exercise increased plasma BDNF on exercising group in a sex difference manner.	Executive processes improved pre- post- exercise with a sex-difference effect.
Ruscheweyh et al., 2011	6 months, 3 day/week, 50 min of either moderate walking (at 50–60% HR), or Stretching (at 30–40% HR); or sedentary control.	62 older adults – age between 50 and 72 years.	Changes in exercise intensity trend for a positive association with changes in BDNF levels.	Pre- post- memory recall score revealed significant main effect of TIME with exercise groups showing better performance after intervention.
^∗^Anderson-Hanley et al., 2012	3 month of either Ergometric cycling, a virtual reality cycling (cybercycling); 3 day/week at 60% of HR.	79 older adults (62 women) – age between 58 and 99 years.	Cybercyclists experienced greater increases of BDNF than traditional cycling.	Cybercycling yielded a medium average effect size for the executive functioning over and above the average effect for traditional exercise, experiencing a 23% reduction in risk of clinical progression to MCI.
Suzuki et al., 2013	6 month of a program including 90-min sessions with aerobic and strength exercises, plus balance and dual-task training, twice a week.	100 subjects with MCI – age between 65 and 95 years.	BDNF yielded positive effect on cognition in older adults with MCI. Higher levels of BDNF at baseline were related better improvements in Alzheimer scale post-intervention.	MCI presented a significant positive effect in memory scores over time with exercise, while controls decreased in memory score after intervention.
^∗^Leckie et al., 2014	Participants started walking for 10 min and increased 5-min weekly until 40 min per session along a total of 12 months, at a target zone of 60–70% of HR.	90 subjects (57 women) – age between 55 and 80 years.	Older ages were associated with lower levels of BDNF at baseline. There was a significant interaction between age and exercise-related changes on serum BDNF.	There was an interaction between age and exercise group in the improvements of executive parameters with younger individuals achieving better scores.
^∗^Nascimento et al., 2014	Multimodal exercise program, 3 /day week, 60 min a session, during 16 weeks. Intensity not declared.	67 subjects (44 women) divided in: with MCI (*n* = 20) and without MCI (*n* = 15) groups, plus paired controls.	Baseline values of BDNF did not differ between MCI and healthy groups, but significant increases post-intervention were only for trained groups.	Only MCI individuals from exercise group yielded increase in MoCA scores, with main changes were observed for domains of executive functions.
^∗^Vaughan et al., 2014.	16-week multimodal exercise program, 2/day week, 60 min a session including progression and variation, during 16 weeks.	49 female subjects – age between 65 and 75 years.	Exercising group levels of BDNF increased while control’s decreased pronouncing a significant difference, with a large effect.	All the evaluated parameters of executive function showed significant improvement in exercise vs. control individuals, with a moderate to large effect.

*^∗^Final included studies from accurate search.*

Retrieved articles from all databases were referenced in Mendeley (Version 1.17-dev1). Searches were conducted from April 12 to December 06, 2016. All papers were screened by title and full text for possible inclusion, after duplicates removed. Studies from the accurate search should fully meet inclusion criteria comprising: interventions applying at least one session of exercise, with pre- post- measures of BDNF, and reported parameters of EFs (i.e., CF, IC, and WM), in aging subjects with cognitive impairment or dementia. Non-human studies, reviews, letters, case-reports, cross-sectional studies, articles not written in English, as well as studies involving psychiatric, metabolic or movement disorders were excluded. The co-author came up with the same selection of studies independently after screening the literature.

## Results

Retrieved papers revealed a larger amount of studies referring to the effects of exercise either on BDNF production, or on cognitive processing, in populations with metabolic syndromes, psychiatric disorders and some neurological diseases. Data from included studies showed exercise paradigms applied to healthy aging or cognitively impaired individuals among experimental and control groups.

Parameters of spatial attention, engagement and disengagement, WM, and CF were the often measured across studies. Neuropsychological tests comprised Trial making test (TMT) A and B, commonly used for estimating attention and CF; The Stroop Color and Word tests, for evaluating selective attention, CF, cognitive inhibition, and processing speed; Task Switching test, mainly for CF measure; Verbal Fluency test, used for executive control; and the Symbol-Digit Modalities test, which assesses key functions underlying tasks of attention, visual scanning, and motor speed (Monsell, 2003; Tombaugh, 2004; Sheridan et al., 2006; Shao et al., 2014; Rivera et al., 2015)

In the study by Baker et al. (2010), subjects with mild cognitive impairment (MCI), were speared into either high-intensity AE or stretching control group to complete 6 months of exercising 45 – 60 min/day (at 75–85% HR reserve) 4 days/week, or carrying out stretching activities. TMT A and B, Stroop Color and Word, Task Switching, Verbal Fluency and SDM tests were performed in order to evaluate parameters of executive functioning, and serum BDNF analysis were taken before and after the exercise program. Improvements pre- post- intervention were evident for exercise group on executive control, CF, information processing, and selective attention (*P* = 0.04), except for men on the Stroop performances (*P* = 0.86). Moreover, a larger improvement on executive functioning was observed for women vs. men (*f* = 0.72 vs. *f* = 0.33, respectively), in which they associated with gains on fitness (*P* = 0.05). Such effects could not be found within the 3 months of program that included graded intensity enforcement. Total plasma BDNF levels tended to be higher for women than men of the exercise group at baseline (*P* = 0.09).

In Anderson-Hanley et al. (2012) trail, a group of older adults named ‘cybercyclist’ accomplished cycling sessions in which they competed with a “ghost” rider projected in a 3D virtual reality. Sessions of stationary cycling comparable by frequency, intensity, and duration were used as control condition. Blood analyses of BDNF and parameters of EF test by TMT, Stroop Color, and Digit Back- ward techniques were reported pre- post- 3 months intervention. Cybercyclists yielded greater increases in BDNF concentrations than stationary cyclists (*p* = 0.05). A significant difference between groups in changes in EF parameters over 3 months revealed a larger effect for cybercycling (*p* = 0.002, power = 0.93). TMT (*p* = 0.01) and Stroop C (*p* = 0.05) performances were improved only for cybercyclists. Meanwhile, a decline in Digits Back-ward performance was observed for stationary cyclists (*p* = 0.01). Altogether, cybercyclists yielded a medium effect size for EF that was over-and-above the average effect on stationary cycling (*d* = 0.50).

In Vaughan et al. (2014) study, a group of older women were randomly engaged into either an exercise program with 60 min/day that included cardiovascular, strength, and motor control training, or a sedentary control group, during 4 months. BDNF analysis, as well as TMT, Verbal Fluency, Stroop, Digit Back- ward, and a reaction time test were conducted before and after the period. The exercise group yielded better performances in all executive parameters compared to baseline (*p* < 0.03), as well as significant increase in BDNF levels relative to baseline, whereas the sedentary group presented a decrease (*p* < 0.05). Exercising exerted a large effect size for BDNF, and a moderate to large effect size for the psychometric parameters.

In Nascimento et al. (2014) study, older individuals with and without MCI were divided into four groups: two training groups (with and without MCI) and two sedentary control (with and without MCI). Montreal Cognitive Assessment (MoCA) was used for psychometric evaluation and blood BDNF were analyzed before and after a multimodal AE program 1h/session 3 days/week, during a 4 month period. Significant increases in BDNF levels were observed in both exercising groups (*p* < 0.001), but not for control groups. MoCA scores from the exercise and control MCI groups were lower than from the healthy conditions (*p* < 0.001), however, subjects with MCI who exercised displayed significant improvement in MoCA scores (*p* = 0.03), with the changes being mainly observed for the parameters of EF (*p* = 0.05), and attention/concentration (*p* = 0.02) domains.

In Leckie et al. (2014) study, older adults were randomly assigned for a 40 min daily walking (up to 75% of HR), or a stretching program, during 12 months. Task-switching and BDNF parameters were evaluated before and after the period. It was revealed that older ages were associated with lower levels of BDNF at baseline (*p* = 0.001). While the stretching group presented a post-intervention decline in BDNF levels, a positive linear relationship between age and the gains of BDNF was showed by the exercise group. An age-related reduction from baseline executive performances was also negated by participating in the exercise program, as it was shown by partial eta square (*p* = 0.025; $\eta_{p}^{2}$ = 0.064). Exercising exerted a compensatory rule on the age-related reduction of BDNF (*F* = 5.45; *p* = 0.022; $\eta_{p}^{2}$ = 0.060). Further inspection also revealed that serum BDNF levels mediated the relationship between exercise group and task-switch performance specifically for older aged individuals. Exercise- dependent changes in serum BDNF mediated the task performance, specifically for the older participants.

## Discussion

Analyzing included studies it becomes evident the studies differ in methodological sets and design. Studied populations displayed different ages and neuropsychological conditions. Exercise interventions were set in long-term designs with weekly sessions lasting a minimum of 2 months, in spite of this; there could be observed standardized procedures for measuring executive functioning, and a high homogeneity in terms of metabolic systems assessed among the exercise approaches (**Table 1**). Therefore, it was possible to control parameters, as exercise volume and its effects on BDNF concentrations, for discussing their dynamics within the executive processes in the subjects.

The hypothesis that larger demands of exercise-dependent BDNF increase are necessary to pronounce changes at the executive levels was in coherence with the reviewed studies, and in line with the wider literature discussing the effects of exercise on BDNF or EF individually. General outcomes confirmed that both BDNF and psychometric parameters are positively affected by AE, regardless of the previous condition of population. The combined increase of BDNF with EF parameters were observed in all studies reviewed. Unfortunately, the statistical correlation between the two parameters is still the question for a further study.

Positive effects of AE on cognition include a variety of changes in brain processing, often expressed by memory task performances, associated with increases in the size of its critically related structures, such as the hippocampus (Rothman et al., 2012). Changes observed in hippocampus structure accompanied by behavioral responses on cognition have showed the BDNF as the main exercise-related factor involved in the improvement of cognitive-related processes, among different ages and population conditions (Fernandes et al., 2016; Öhman et al., 2016).

The effects of exercise-dependent BDNF have been elucidated at cellular and molecular levels on synaptic plasticity and maintenance, at areas and circuitry of cognitive and executive processing support (Lista and Sorrentino, 2010). BDNF plays the role of modulating signaling processes via interaction with its high affinity tyrosine kinase B receptor (TrkB), broadly expressed across central nervous system. BDNF-TrkB system then activates several cascades of events ultimately promoting the expression of proteins related to neuronal differentiation and survival (Rafieva and Gasanov, 2016). Thereby, the BDNF up-regulation, strongly evoked by exercise, potentiate the protection of neurons from eventual damage, and favor neurogenesis and plasticity (Budni et al., 2015).

Further, the metabolic loads requested by exercise may be implicated in the magnitude of BDNF responses. For instance, in study by Ruscheweyh et al. (2011), a trend for a positive association between the level of intensity and blood BDNF of old subjects who exercised for 6 months was revealed along with a significant main effect for the episodic memory performances. In coherence, memory improvements as well as a reduction of the risk for Alzheimer’s were found in association with increases in serum BDNF within 6 months of a multimodal exercise program for individuals with older ages (Suzuki et al., 2013). It is believed that the higher enhancements of BDNF disposal related to larger volumes of AE are capable of counteract both cognitive decline and the risk of dementia (Paillard, 2015).

In accordance, Leckie et al. (2014) showed that baseline levels of BDNF are lower as individuals advance with age. Nevertheless, such deficits of BDNF could be reversed after the long-term exposure to AE, together with an age- related decline identified for the executive functioning. Those findings indicate that age should not be a determinant factor for the exercise-dependent response of BDNF or its benefits, reinforcing the notion that BDNF-dependent gains at the level of executive circuitry emerge in an exercise dose- dependent manner.

Moreover, it is established that repetitive activation in cortical areas, such as the temporal and pre-frontal lobes, due to exercise challenging evokes adaptive responses at neuronal levels that positively impact on cognitive processes, and thus in executive networks and circuitry (Phillips et al., 2014). Therefore, exercise programs such as walking, a task that lays on higher-order EFs, have demonstrated to be effective on preventing and postponing executive decline in older age individuals, whose changes are more likely evident (Scherder et al., 2014).

The mechanisms of exercise-dependent BDNF enhancement and its repercussion in brain functioning are actively studied in animal models (Chen and Russo-Neustadt, 2010; Sleiman et al., 2016) with positive effects reported on animal cognition in association with exercise-mediated BDNF increases (Bechara et al., 2014). Although, it is difficult to compare exercise-dependent improvements in EF of humans in course of dementia with achieves of animal models of study, as these last do not simulate human state an in satisfactory way, they though allow us to study nervous system responses to exercise in the level of molecular processes.

Brain-derived neurotrophic factor outcomes were shown to be more responsive to exercising than the executive parameters. All individuals exposed to some exercise within studies presented increases in levels of BDNF, in consonance with the wider literature evidence that either an acute or a chronic exposal to AE significantly increase BDNF concentrations for both female and male (Dinoff et al., 2016), with the magnitude of effects possibly enhancing over time (Szuhany et al., 2015). In line with it, Nascimento et al. (2014), demonstrated that while the levels of BDNF significantly increase for both healthy and MCI subjects exposed to exercise, individuals with MCI do not present gains on executive parameters comparable to those without. This demonstrates individuals with some cognitive impairment should count on larger doses of exercise to achieve similar responses on BDNF-related executive processes than those displaying a natural aging decline.

In addition, the finding of Anderson-Hanley et al. (2012) study converges to the thesis that the executive processing is not only affected by exercise-related BDNF support. Instead, the executive circuitry is also responsive to the demands of cognitive stimulation involved in exercise processes. They show evidence that BDNF responses to exercise were potentiated by the amount of cognitive stimuli when it was revealed that the cyclist who exercised in a cognitively enriched set of 3D virtual challenge yielded greater increases of BDNF than control cyclists exposed to the same exercise volume. As expected, the group with greater gains on BDNF also presented greater performances of EF.

According to Baker et al. (2010), sex differences in studied individuals were observed with women yielding higher improvements on EF. Although the difference of BDNF at baselines have not reached statistical significance, a trend was found only for those presenting better executive responses, accompanied by improvements on fitness status. As this observation was described only in the one study, that fact must be taken into account and the presence and evidence of such differences must be carefully analyzed in future.

Physical and cognitive stimulation individually, have been shown to specifically benefit older adults on different cognitive domains and competences, although with limited effects over global cognition. Therefore, a combination of both should suit as a powerful strategy to overcome this shortcoming (Bamidis et al., 2015). The increase of blood flow and BDNF expression pronounced by the physical stress of exercising, combined with the hemodynamic request driven by the amount of cognitive stimuli must direct the neurotrophic support to the circuits engaged on the task (Whiteman et al., 2016).

Positive correlations were consistently observed between the effects of exercise on both BDNF and executive functioning parameters across studies, and supported by the wider literature individually exploring the effects of exercise in such variables. We moreover believe AE is effective in protecting the executive competences from a decline in a manner that associate volume (time of exposure and intensity), and lots of cognitive engagement. While exercising physiologically prepares the brain to respond to a cognitive stimulus, the cognitive stimulation drives neural requirements to the specific circuitry involved in its support.

It is plausibly assumed that executive processes may require larger amounts of exercise-dependent BDNF support than what is commonly achieved through acute experiments at memory and cognitive processes, especially for individuals under pathological processes of cognitive decline. Nevertheless, such difference could be minimized though the enrichment of exercise volume and cognitive stimulation.

Importantly, a statistical evaluation of the dynamics of BDNF and EF parameters, with regard to possible sex difference should be performed in order to provide tools for the development of individual AE approaches in clinical neuropsychology.

To our knowledge this is a first study that investigates the nuances of the modulatory effects of exercise on executive capabilities, dependent on integrity of wider schemes of brain networks, through regulation of the synthesis of BDNF.

## Limitation

Exercise influence on cognition is still a poorly explored field in the context of aging and dementia. Although it was noticeable by the studies included in this review that exercise has a positive effect on EF and BDNF, this does not necessarily mean that enhancing BDNF release is the only mechanism by which exercise is responsible for cognitive benefits. Further experimental research should concern better control of the exercise metabolic targets, as well as provide a standard data presentation toward facilitating analysis between changes in EF and BDNF level, as so far most of the studies have not performed any correlational analysis. Due to limited available literature found of studies with humans and the evident difficulties in molecular pathway study within humans, further studies should also consider including findings from adequate animal models for a more extensive data comparison.

## Author Contributions

KMA contributed to the study on the original idea and design, interpretation of data and approval of the final version. GMA contributed to the writing of the article and the acquisition of data and interpretation of data.

## Conflict of Interest Statement

The authors declare that the research was conducted in the absence of any commercial or financial relationships that could be construed as a potential conflict of interest.
